# Roles of Neutrophils in Glioma and Brain Metastases

**DOI:** 10.3389/fimmu.2021.701383

**Published:** 2021-08-13

**Authors:** Ya-Jui Lin, Kuo-Chen Wei, Pin-Yuan Chen, Michael Lim, Tsong-Long Hwang

**Affiliations:** ^1^Department of Neurosurgery, Chang Gung Memorial Hospital, Linkou, Taiwan; ^2^Graduate Institute of Natural Products, College of Medicine, Chang Gung University, Taoyuan, Taiwan; ^3^Department of Neurosurgery, Stanford University School of Medicine, Stanford, CA, United States; ^4^Department of Neurosurgery, New Taipei Municipal TuCheng Hospital, Chang Gung Medical Foundation, New Taipei, Taiwan; ^5^School of Medicine, Chang Gung University, Taoyuan, Taiwan; ^6^Department of Neurosurgery, Chang Gung Memorial Hospital, Keelung, Taiwan; ^7^Research Center for Chinese Herbal Medicine, Research Center for Food and Cosmetic Safety, and Graduate Institute of Health Industry Technology, Chang Gung University of Science and Technology, Taoyuan, Taiwan; ^8^Department of Anesthesiology, Chang Gung Memorial Hospital, Taoyuan, Taiwan; ^9^Department of Chemical Engineering, Ming Chi University of Technology, New Taipei City, Taiwan

**Keywords:** neutrophils, gliobastoma, brain metastases, neutrophil extracellular traps, nanocarrier

## Abstract

Neutrophils, which are the most abundant circulating leukocytes in humans, are the first line of defense against bacterial and fungal infections. Recent studies have reported the role and importance of neutrophils in cancers. Glioma and brain metastases are the most common malignant tumors of the brain. The tumor microenvironment (TME) in the brain is complex and unique owing to the brain-blood barrier or brain-tumor barrier, which may prevent drug penetration and decrease the efficacy of immunotherapy. However, there are limited studies on the correlation between brain cancer and neutrophils. This review discusses the origin and functions of neutrophils. Additionally, the current knowledge on the correlation between neutrophil-to-lymphocyte ratio and prognosis of glioma and brain metastases has been summarized. Furthermore, the implications of tumor-associated neutrophil (TAN) phenotypes and the functions of TANs have been discussed. Finally, the potential effects of various treatments on TANs and the ability of neutrophils to function as a nanocarrier of drugs to the brain TME have been summarized. However, further studies are needed to elucidate the complex interactions between neutrophils, other immune cells, and brain tumor cells.

## Introduction

Paul Ehrlich coined the term neutrophils, which are also known as polymorphonuclear cells, in the late nineteenth century due to their preferential uptake of neutral pH dye and their inability to retain acidic or basic dyes (1, 2). Neutrophils, which are the most abundant circulating leukocytes in mammals, have a short life span and the neutrophil population is constantly replenished from the bone marrow (3). The first line of defense against bacterial and fungal infections is neutrophils. Additionally, neutrophils can modulate the adaptive immune response at the inflammation site through interaction with antigen-presenting cells and lymphocytes (4–6). Since the nineteenth century, inflammation has been correlated with the pathogenesis of cancer (7). Cancer-related inflammation, a hallmark of tumor biology, involves both stromal and inflammatory cells in the tumor microenvironment (TME) (8, 9). The immune privilege of the central nervous system (CNS) can be attributed to the lack of dedicated lymphatic channels in the past (10). In 2015, a novel route of lymphatic channels from the brain that runs parallel to the dural venous sinuses and travel to the deep cervical lymph nodes was identified (11).

In the 1890s, William Coley first conceived the concept of cancer immunotherapy. Ehrlich and Thomas and Burnet further developed this concept in the 1950s and 1960s, respectively (12–15). Cancer immune surveillance involves the detection and elimination of the tumor cells by the immune system (12, 15). Thus, cancer immunotherapies, especially immune checkpoint inhibitors (ICIs), are an emerging therapeutic modality and have revolutionized cancer management (16, 17). In contrast to other immune cells, such as macrophages, neutrophils were not considered to be major players in the TME. However, recent developments in genetic tools have revealed the importance of neutrophils in the TME, especially in tumor progression and tumor resistance (2, 18). The presence and phenotype of neutrophils in the TME determine the efficacy of both traditional and novel cancer therapies (3, 19).

As neutrophils play an important role in the TME, they are potential targets to enhance the efficacy of immunotherapy. However, the role of neutrophils in brain cancer biology remains unclear. In this review, we focus on the role of neutrophils in glioma and brain metastases (BMs), which are the most common malignant brain tumors. This review proposes the neutrophil-to-lymphocyte ratio (NLR) as a biomarker for brain cancer. Additionally, this review discusses the heterogeneity of tumor-associated neutrophils (TANs). Furthermore, the current therapeutic modalities using neutrophils as targets or carriers to treat glioma and BM have been summarized.

## Origin, Life Cycle, and Biological Functions of Neutrophils

More than 10^11^ neutrophils are produced every day under steady-state conditions (20, 21). Emergency granulopoiesis is a process in which the formation of neutrophils rapidly increases in response to infection or cancer (22).

### Granulopoiesis

**Figure 1:**
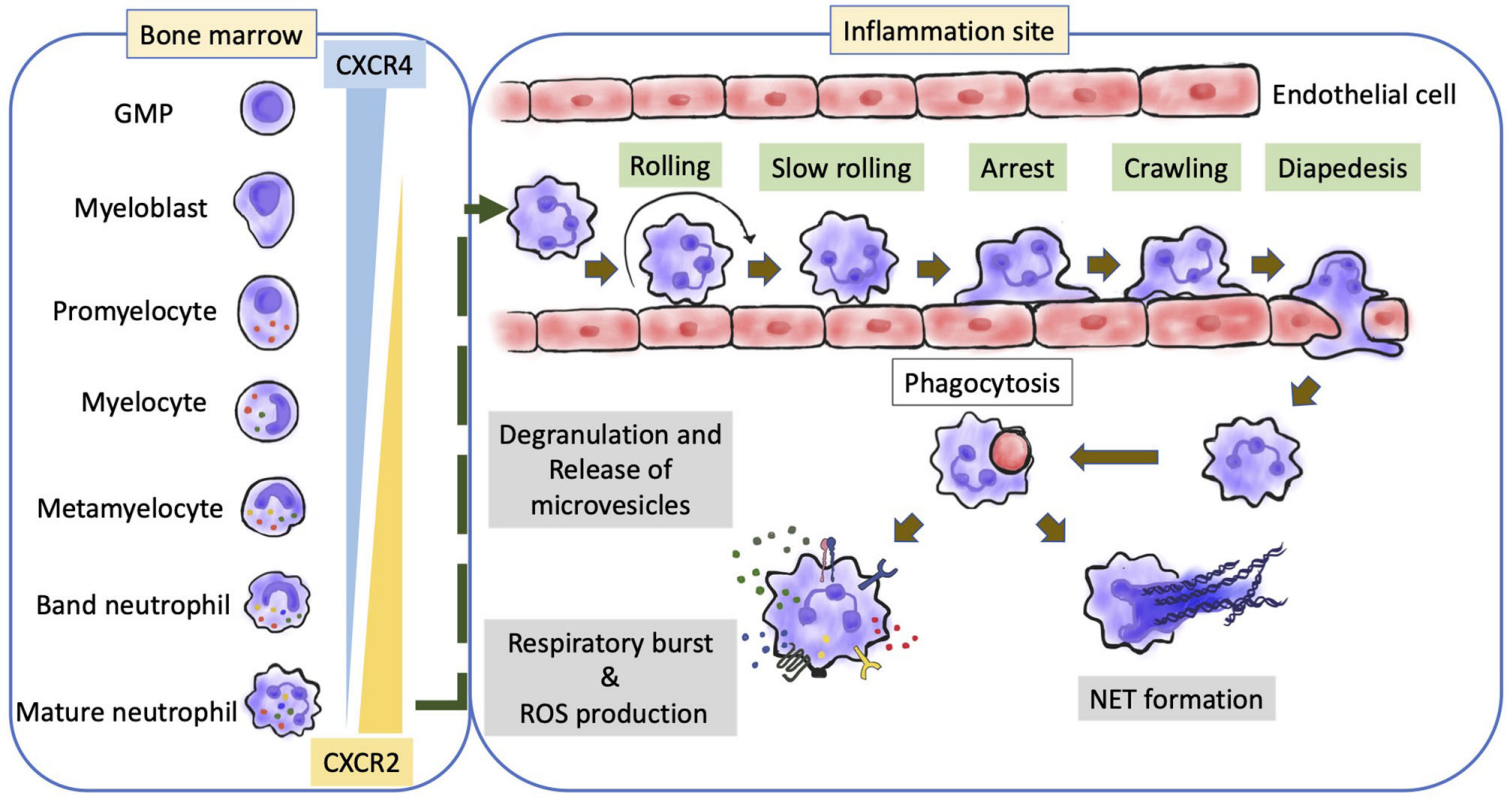
Overview of neutrophil development and biological function. The production and maturation of neutrophils develop in bone marrow. After stress or inflammatory stimulation, neutrophils undergo a special recruitment cascade and eventually migrate into inflammatory tissues. Respiratory burst, degranulation, and NETs formation are the main mechanisms responsible for inflammation, leading to the elimination of the invading microorganisms or promoting the inflammatory response; CXCR, CXC-chemokine receptor; GMP, granulo-monocytic progenitor; NETs, neutrophil extracellular traps.

Neutrophils are generated from lymphoid-primed multipotent progenitors (LMPPs) (23), which are derived from hematopoietic stem cells. LMPPs further differentiate into granulocyte-monocyte myeloid progenitors (24, 25). Neutrophils undergo maturation in the following steps: the formation of myeloblasts, followed by the formation of promyelocytes, myelocytes, metamyelocytes, band neutrophils, and segmented neutrophils (**Figure 1**) (22, 26, 27). The transitions from myeloblasts to promyelocytes, and from myelocytes to metamyelocytes are characterized by the appearance of primary granules and secondary granules respectively. Meanwhile, the transition from the band neutrophils to the segmented neutrophils is characterized by the formation of tertiary granules (21, 28). These granules comprise various defensive factors and enzymes, such as myeloperoxidase (MPO), elastase, defensins, cathelicidins, and matrix metalloproteinases (MMPs), which protect against opportunistic infections and mediate the alleviation of inflammation (28, 29).

Granulocyte colony-stimulating factor (G-CSF), which is the master regulator of neutrophil generation and differentiation (30–32), can induce the proliferation and differentiation of myeloid progenitors. The expression of G-CSF receptor (G-CSFR) is detected in the myeloid lineage from the early stem and progenitor cells to fully differentiated neutrophils (33, 34). G−CSFR-signal transducer and activator of transcription 3 (STAT3) signaling regulates neutrophil formation (35). Other molecules, such as granulocyte-macrophage colony-stimulating factor (GM-CSF), interleukin 6 (IL−6), and KIT ligand (also known as KITLG) are reported to be involved in granulopoiesis (36–38). For example, these cytokines are upregulated in several animal models of cancer, which results in enhanced granulopoiesis and neutrophilia (39–45).

### Neutrophil Retention and Release From the Bone Marrow

In contrast to other immune cells, neutrophils are released from the bone marrow as terminally differentiated mature cells. Under homeostatic conditions, approximately 1%–2% of all neutrophils are mature neutrophils that circulate throughout the body (46). The immature cells are retained in the bone marrow due to the interaction between C−X−C chemokine receptor 4 (CXCR4) and CXCR2 (**Figure 1**). Osteoblasts and other bone marrow stromal cells constitutively express CXCL12 and tether CXCR4+ neutrophils (immature) in the bone marrow. Meanwhile, mature neutrophils exhibiting CXCR2 expression are released into the circulation through the interaction with CXCL1 and CXCL2 secreted by the endothelial cells and megakaryocytes (47–52). Several adhesion molecules, such as integrin subunit α4 (ITGα4), vascular cell adhesion molecule 1 (VCAM1), and some proteases also play a critical role in neutrophil retention (52–55). G−CSF is reported to be a disruptor of neutrophil retention (56).

The molecules that regulate neutrophil release into the circulation are upregulated in tumors. These molecules override the neutrophil retention signals in the bone marrow to facilitate neutrophil egress and consequently increase neutrophil counts (39–42, 57). In addition to cytokines derived from tumor cells (41, 42, 57), stromal and immune cells can upregulate the expression of these molecules in tumor-bearing mice. The aberrant production of cytokines by the tumors or stromal cells can cause an imbalance in neutrophil retention and release from the bone marrow.

### Biological Functions

Neutrophils regulate immunity and inflammation by recognizing and responding to microbial and inflammatory stimuli (58, 59). Phagocytosis and enzymatic processes (**Figure 1**), including the generation of reactive oxygen species (ROS) through the NADPH oxidase 2 (NOX2) and the release of granule-derived MPO, hydrolytic enzymes (such as elastase, lysozyme, and MMPs), and other antimicrobial proteins/peptides (such as lactoferrin and defensins) can eliminate the invading microorganisms after pathogen recognition (60, 61). Neutrophil granule-derived molecules can directly kill microorganisms, mediate positive feedback loops in neutrophils, or attract and activate monocytes (62, 63). Classical effector responses interact with each other at several levels, including the primary release of granules into the phagosome, processing of NOX2-derived superoxide and H_2_O_2_ by granule-derived MPO, or NOX2-mediated activation of granule proteins (60–62, 64). Neutrophils also release their DNA into the extracellular space to form neutrophil extracellular traps (NETs), a complex of DNA and neutrophil-derived antimicrobial molecules (**Figure 1**) (65). In addition to trapping and killing bacteria (65), NETs are involved in various inflammatory diseases (66). NET formation (also called ‘NETosis’) is dependent on MPO, neutrophil elastase, peptidylarginine deiminase 4 (PAD4), and gasdermin D, which are potential therapeutic targets for NET-mediated pathologies (67–69). The genetic deficiency of NET-degrading DNases results in the development of NET-mediated vascular occlusion (70). However, previous studies have reported controversial findings on NETs generation and function.

Neutrophils can release various chemokines, cytokines, and lipid mediators through modulation of gene expression although they exhibit limited transcriptional activity (71–73). Therefore, neutrophils are involved in regulating the immune response and the interaction of various immune and non-immune cells (74–76). However, the functional relevance of these neutrophil-derived mediators is not completely understood. Neutrophils also release extracellular vesicles that exhibit antimicrobial functions (77), mediate the effects of neutrophil-derived LTB4, and contribute to the pathogenesis of inflammation and cancer (78–80).

## Current Therapeutic Strategies for Glioma and BMs

Brain tumors are a mixed group of primary and metastatic neoplasms that exhibit varying degrees of malignancy. Although malignant lesions are uncommon, their incidence has increased rapidly in highly developed and industrialized countries (81). Malignant brain tumors directly affect neurological function, psychological health, and quality of life (82). In the last few decades, advances in diagnostic methods, including the development of computed tomography in the mid-1970s and magnetic resonance imaging (MRI) in the mid-1980s, have revealed an increased incidence of brain tumors (83, 84).

Glioma is the most prevalent type of primary malignant brain tumor. Glioblastoma (GBM) is the worst grade of glioma with a median overall survival of 14 to 17 months and a five-year survival rate of only 5.6% despite of the traditional triad of surgical excision, radiotherapy, and chemotherapy (85–88). Recently, tumor-treating fields, which involve the use of low-intensity alternating electric fields to interfere with cell division and organelle assembly, have been demonstrated to extend the OS of patients with glioma to 20.9 months (89). Besides, several studies have focused on immunotherapy as an effective treatment paradigm (90, 91). The results of phase I and II clinical trials on cancer vaccines were encouraging. The synergistic effects of anti-tumor vaccines and standard therapy have been previously demonstrated (92–96). Immunotherapy is believed to be a promising approach for enhancing the efficacy of the current chemoradiation regimen. However, phase III clinical studies have revealed the poor efficacy of ICIs and vaccine therapy for GBM. Nivolumab (monoclonal antibody to PD-1) of checkMate 143 (NCT02017717) (97) and Rindopepimut (EGFRvIII specific peptide and KLH conjugate) of ACT IV (NCT01480479) (98) both terminated early because no significant survival benefit. There are several obstacles to the success of GBM immunotherapy, such as the heterogeneity and low mutation burden of the tumors and local/systemic immunosuppression induced by GBM (99–103).

BMs, which occur in 10%–40% of patients with cancer, are the most common malignant brain tumors in adults (104, 105). The increased surveillance and improved control of primary cancer resulting in prolonged survival have increased the incidence of BMs (106). The most common primary sites of BMs are lung and breast carcinomas and melanoma (107). The management of BMs has been revolutionized by improved brain imaging and management of systemic diseases, distribution of stereotactic irradiation, and the development of less invasive surgical techniques that enable the removal of BMs even from eloquent brain areas with minimal morbidity (108). Although whole-brain radiation therapy (WBRT), stereotactic radiosurgery (SRS), and systemic therapies have improved the treatment outcomes, surgical resection is the major therapeutic strategy for large BMs. The indications for resection include a symptomatic mass, a mass with edema requiring high-dose steroids, a mass with a size more than 3 cm, and a mass with an unknown primary cancer (108). In addition, recent advances in immunotherapy have increased the therapeutic options for patients with metastatic melanoma. Ipilimumab (monoclonal antibody to CTLA-4) and nivolumab can stimulate T cell-mediated anti-tumor immune response. Previous studies have reported that ipilimumab treatment achieves CNS disease control in 24% of patients with melanoma exhibiting asymptomatic BM and 10% of patients with the symptomatic disease (109). Additionally, dual immune checkpoint blockade of ipilimumab and niolumab has achieved higher intracranial responses (110). However, the efficacy of other ICIs for BMs has not been examined.

The understanding of the interaction of immune cells in the brain TME can aid in the development of effective immunotherapy strategies. Neutrophils, which are abundant in the circulation, are a potential therapeutic target for BMs as they have critical roles in immunity and inflammation. Therefore, many recent researches focused on the analyses of brain immune TME landscape *via* flow cytometry, RNA sequencing, protein arrays, culture arrays, and spatial tissue characteristics in glioma and BMs (111, 112). Disease-specific immune cells enriched with pronounced differences in tissue-resident microglia, infiltrating monocyte-derived macrophages, neutrophils, and T cells between gliomas and BMs. Abundance of TAMs infiltrated in gliomas, whereas T cells were much fewer. However, lymphocytes and neutrophils accumulated obviously in BMs. This implies that tumors indeed shape their TME while growing within the brain. It is different from cancers that metastasize from extracranial sites. In addition, there were additional differences for BMs originated from distinct primary tumors. For example, abundance of CD4+ and CD8+ T cells represented the major immune compartment in melanoma BMs, whereas it showed the highest neutrophil infiltration in breast BMs. Therefore, defining the specific immunological signature of brain tumors can facilitate the rational design of targeted immunotherapy strategies.

## NLR as a Biomarker

The local inflammatory process in the TME and systemic inflammation play important roles in cancer development and progression (113–115). The hallmarks of cancer-related inflammation include the presence of inflammatory cells and inflammatory mediators (for example, chemokines, cytokines and prostaglandins) in tumor tissues similar to that seen in chronic inflammatory responses (8). The upregulated levels of C-reactive protein, hypoalbuminemia, some cytokines, and leukocytes and their subtypes in the blood can be quantified to determine systemic inflammation (116, 117).

Inflammatory cytokines and chemokines, which can be produced by both tumor and stromal cells, contribute to the progression of malignancy (7). The increased NLR has been associated with increased peritumoral infiltration of macrophages and upregulation of several cytokines, such as IL-6, IL-7, IL-8, IL-9, IL-12, IL-17, and interferon-γ (IFNγ) (118, 119). Neutrophils and macrophages are reported to secrete tumor growth-promoting factors, including vascular endothelial growth factor (120, 121), hepatocyte growth factor (122), IL-6 (123), IL-8 (124), MMPs (125), and elastases (126), which promote the development of pro-tumor TME. Nevertheless, IL-12 and IFNγ are reported to enhance antitumor effect. IL-12 can be used as combinatorial immunotherapy and effective in preclinical glioblastoma model (127). On the other hand, cancer-related inflammation, like smouldering inflammation, in the TME has many pro-tumor effects. It exacerbates progression of malignant cells, promotes angiogenesis and metastasis, suppresses adaptive immune responses, and alters responses to hormones and chemotherapeutic agents (8). Therefore, the cons and pros of inflammation in cancer are still needed to be elucidated in the future.

Thus, biochemical markers of inflammation in patients with various cancers have been incorporated into prognostic scores (128). Neutrophilia, an inflammatory response, inhibits the immune system by suppressing the cytolytic activity of immune cells, such as cytotoxic T cells and natural killer (NK) cells (129, 130). Several studies have reported the critical roles of tumor-infiltrating lymphocytes (TILs) in cancer. The upregulation of TILs has been associated with improved treatment response and prognosis (131–133). Therefore, an elevated NLR in peripheral blood was recognized as a poor prognostic indicator recently (134). The mechanisms underlying the correlation between high NLR and poor prognosis in patients with cancer have not been elucidated.

Recent studies have developed sensitive and accurate biomarkers for cancer. The changes in the blood NLR can be used to determine the optimal therapy for patients with advanced-stage cancer (135, 136). The prognostic value of NLR in early-stage cancer is lower than that in patients with advanced-stage cancer. However, NLR can aid in the evaluation of the early effects of systemic therapy (137–140). Some small-scale studies have reported that chemotherapy improved the clinical outcomes of patients with cancer through the alleviation of dysregulated NLR (135, 136).

Most patients with glioma exhibit neutrophilia (141) due to the increased production of G-CSF by tumor cells to promote growth (142, 143). G-CSF shifts bone marrow hematopoiesis toward the myeloid lineage and away from the lymphocyte lineage. This leads to a high NLR in patients with glioma. Several studies have demonstrated that the NLR of more than 4 is associated with poor prognosis when examined before treatment (144, 145), after the second surgery (146), and after TMZ and RT therapies (147). NLR of less than 4 is associated with improved prognosis in GBM with wild-type IDH1 (148). The baseline neutrophil count in the blood can also predict bevacizumab efficacy in patients with GBM (149). The upregulated surface expression of CD11b, a marker of neutrophil activation, can be used as an early predictor of tumor progression in patients with GBM (150). Arginase I produced by circulating neutrophils promotes tumor growth and induces immunosuppression (151).

The OS of patients with BMs is poor (152). Therefore, there is a need to develop multidisciplinary and individualized treatment approaches for BMs. Prognostic models with recursive partitioning analysis and graded prognostic assessment that incorporate multiple patient factors have been established to predict the treatment outcomes (153, 154). Although patients exhibit prolonged survival and undergo novel therapies, including targeted and immune therapy with SRS, novel factors must be incorporated into the predictive system to examine the survival benefits of novel therapies. Recently, NLR has been identified as one of the markers for predicting the outcomes of novel therapies (9, 155). The inflammatory cells are reported to cross into the brain (11). Hence, the association between NLR and OS in patients with BM was investigated. Several studies have investigated the optimal dichotomous cutoff values of NLR in various BMs. NLR values of more than 5, 3.3, and 6 are correlated with poor outcomes of post-resection, pre-SRS, post-SRS, respectively (156–158). There were no common optimal dichotomous cutoff values for NLR in all studies. The optimal NLR to predict OS in various malignancies ranged from 2.18 to 7.5 (144, 147, 159–161). The increased NLR value is reported to be correlated with a high risk of local recurrence. Patients with elevated NLR are likely to exhibit neuronal death (157). Prospective validation must be performed to validate the simple and systemic marker NLR for determining if aggressive treatment is needed following initial treatment. Further studies are needed to examine the interaction between BMs and immunological environments.

Thus, NLR has been proposed as a surrogate marker for the prognosis of various cancers as lymphocyte and neutrophil counts can be easily determined based on the complete blood count. However, acute conditions, such as bacterial or viral infections or drug treatments may overlap chronic ongoing inflammation and affect the neutrophil and lymphocyte counts. Hypertension, diabetes mellitus, metabolic syndrome, left ventricular dysfunction and hypertrophy, acute coronary syndromes, cardiovascular diseases (162, 163), abnormal thyroid function tests, renal or hepatic dysfunction, previous history of infection (<3 months), inflammatory diseases (164), and some medications (e.g. steroids) can potentially affect the measurement of NLR. Therefore, NLR must be carefully validated.

## TAN Heterogeneity and NETs

**Figure 2:**
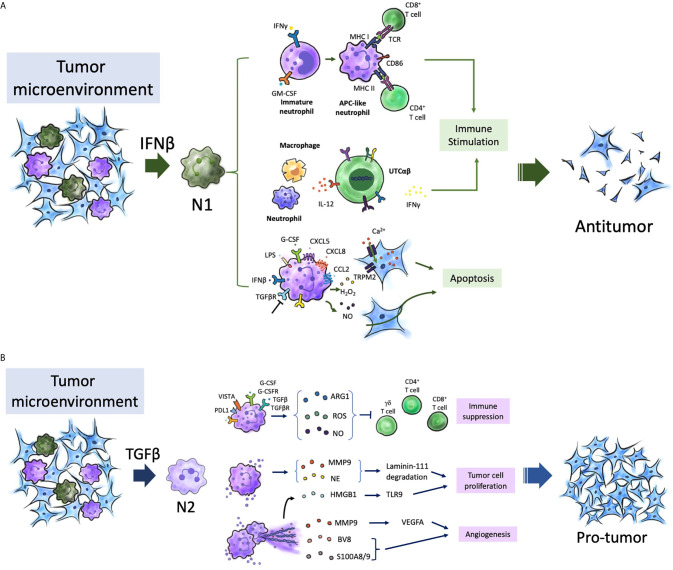
Neutrophils in tumor microenvironment. TANs participate in different stages of tumorigenesis, and are polarized into N2 (pro-tumor) in the presence of TGF-β, and polarized into N1 (antitumor) in the presence of IFN-β in the TME. **(A)** APC-like neutrophils from immature neutrophils with presence of GM-CSF and IFNγ express MHC I/II and the co-stimulatory molecules CD86, 4-1BB ligand (4-1BBL) and OX40 ligand (OX40L), which enhance T cell immunity. UTC_αβ_, essential for effective antitumor immunity, can be polarized to a type 1 immune response and IFNγ production through IL-12 production from macrophage which was amplified by neutrophils. Different stimuli (G-CSF) and chemokines of CXCL5/8, and CCL2, LPS, and IFNβ promote an oxidative burst and the production of hydrogen peroxide (H_2_O_2_), as well as blocking TGFβ pathway. Collectively, H_2_O_2_ triggers the activation and opening of TRPM2, and further leads to a lethal influx of calcium (Ca^2+^) into tumor cells. **(B)** N2 phenotype promoted the tumor growth, angiogenesis, and immunosuppression. Neutrophils express immune check point receptor of PDL1 and VISTA. They also have been shown expression of ARG1, ROS, and NO in the presence of G-CSF and TGFβ in TME, which inactivate T cells. HMGB1 from the release of NETs can activate TLR9-depedent pathway which sustain tumor cells proliferation. NE and MMP9 cleave laminin-111, and then cleaved laminin-111 triggers the proliferation of tumor cells through activation of integrin signaling. Neutrophils promote angiogenesis through the release of the pro-angiogenic factors BV8, S100A8/9, and MMP9 that activate VEGFA. TANs, tumor-associated neutrophils; IFN-β, Interferon-β; TGF-β, transforming growth factor-β; TME, tumor microenvironment; GM-CSF, granulocyte–macrophage colony- stimulating factor; IFNγ, Interferon- γ; CXCL5/8, CXC-chemokine ligand 5/8; CCL2, CC-chemokine ligand 2; LPS, lipopolysaccharide; TRPM2, transient receptor potential cation channel, subfamily M, member 2; UTC_αβ_, CD4^–^CD8^–^TCRαβ^+^ double-negative unconventional T cells; PDL1, programmed cell death 1 ligand 1;VISTA, and V-domain immunoglobulin suppressor of T cell activation; Arg-1, arginase 1; ROS, reactive oxygen species; NO, nitric oxide; G-CSF, granulocyte colony-stimulating factor; HMGB1, high mobility group protein 1; NETs, Neutrophil extracellular traps; TLR9, Toll-like receptor 9; NE, neutrophil elastase; MMP9 matrix metalloproteinase 9; VEGFA, vascular endothelial growth factor A.

The complex roles of neutrophils in both tumor growth and metastasis have been reported. The role of neutrophils in cancer is dependent on various factors and may result in a pro-tumoral or an antitumoral effect (2). TANs in the TME and the functions of neutrophils, including circulating and bone marrow neutrophils and neutrophil lineage cells, determine their role in cancer. TANs are polarized to anti-tumor (N1) or pro-tumor (N2) phenotypes (**Figure 2**) (165). Since 2015, the functional plasticity of TANs and their ability to undergo alternative activation upon exposure to various cues in the TME have been demonstrated (166, 167). For example, the presence of transforming growth factor-β (TGFβ) promotes a pro-tumor phenotype (N2 TANs), whereas the presence of IFNβ or the inhibition of TGFβ signaling promotes the anti-tumor phenotype of TANs (N1 TANs) (165, 168). The binary N1/N2 phenotypes maybe an oversimple classification system to describe neutrophil polarization, as well as the M1/M2 phenotypes for TAMs (169–171). Neutrophil polarization may involve a spectrum of activation states. Multiple heterogeneous neutrophil subsets have been observed in the circulation of both tumor-bearing mouse models and patients with cancer. The following three distinct neutrophil populations have been identified in the circulation of both patients with cancer and mouse models: mature high-density neutrophils (HDNs), mature low-density neutrophils (LDNs), and immature LDNs (172–175). Mature HDNs exhibit an anti-tumor N1-like phenotype. However, mature LDNs, which are not cytotoxic, exhibited impaired functions and immunosuppressive properties (172–174).

The dual and opposite functions of neutrophils in tumor immunity may vary during the progression of cancer (173). Neutrophils can influence tumor development by modulating the recruitment, profile and phenotype of other immune cells, especially TAMs and tumor-infiltrating lymphocytes (TIL) which are major components of the immune TME. Neutrophils are reported to exhibit growth-inhibitory activity against early-stage cancers (176–178). Neutrophils are involved in networks of T cell-dependent antitumor immunity. In TME, with the contribution of IFNγ and GM-CSF, immature neutrophils become mature and have the function of antigen-presenting cells (APCs). Then, these APC-like neutrophils stimulate the proliferation of both CD4+ and CD8+ T cells through the major histocompatibility complex (MHC) class I and class II molecules and the co-stimulatory molecules CD86, 4-1BB ligand (4-1BBL) and OX40 ligand (OX40L) expression (177, 178). Furthermore, chemokine including CXCL10, CCL2, CCL3, CXCL1 and CXCL2 produced by TANs could recruit T cells as well as other leukocytes (74, 179, 180). Neutrophils are also able to kill tumor cells through direct contact and *via* ROS generation (165, 176, 181). The transient receptor potential cation channel, subfamily M, member 2 (TRPM2), a H_2_O_2_-dependent channel, can induce influx of calcium into tumor cells and further lead to cell death. In addition, TNF-related apoptosis inducing ligand (TRAIL) and TNF can induce the expression of the hepatocyte growth factor receptor (HGFR; also known as MET) on neutrophils (182). HGF present in the TME induce neutrophil recruitment and production of nitric oxide (NO), which results in killing of tumor cells (182). Neutrophils also engage in an interaction between macrophages and a subset of unconventional T cells (UTCs), known as CD4^–^CD8^–^TCRαβ+ double-negative UTCs (UTC_αβ_). The UTCs were essential for effective antitumor immunity (179). Neutrophils are able to enhance the production of IL-12 by macrophages, and then promote UTC_αβ_ polarize towards a type 1 immune response and IFNγ production (179). However, the roles, mechanisms, significance of UTC_αβ_ in human tumors still need to be elucidated.

In contrast, the strong immunosuppressive activity of neutrophils is associated with polymorphonuclear myeloid-derived suppressor cells (MDSCs) in advanced-stage cancer. Polymorphonuclear MDSCs also share features with immature neutrophils. Recently, early neutrophil progenitor was reported to exhibit pro-tumor activity (173, 177, 183). The disease stage, the tumor type, and tissue context are all key determinants of neutrophils in promoting or restraining cancer. Neutrophils with intrinsic anti-tumor activity are recruited to the tumor, where they are reprogrammed to an immunosuppressive pro-tumor phenotype (from N1 to N2) (165, 173).

The ability of cancer-related neutrophils to release NETs has piqued the interest of the scientific community. NETs, which are released from neutrophils in response to extracellular pathogens, typically comprise fibrous decondensed chromatin with associated histones, MPO, and various cytoplasmic proteins, such as neutrophil elastase, cathepsin G, and lactoferrin. Although NETs are reported to be released in response to cancer cells, their role in cancer is not clear.

NETs, the release of ROS, and the trapping of cancer cells could theoretically promote a cytotoxic effect and inhibit the dissemination of cancer cells (184, 185). However, most studies have only described this phenomenon in circulating neutrophils (186–188). Some studies have also reported that NETs are spontaneously produced in samples from patients with cancer and that NETs promote metastatic dissemination after surgical stress (187). In mouse models, sustained lung inflammation caused by tobacco smoke exposure or nasal instillation of lipopolysaccharide leads to NETs formation (189). Neutrophil elastase and matrix metalloproteinase 9 (NET-associated proteases) cleave the extracellular matrix protein laminin. The proteolytically remodeled laminin induced proliferation of dormant cancer cells by activating integrin α3β1 signaling. Then, the dormant cancer cells are awakening to aggressively growing metastases. Therefore, NETs are hypothesized to act within the primary tumor to promote disease progression and dissemination. The enhanced release of NETs by the neutrophils has been suggested to promote tumor progression and metastatic dissemination. Thus, further studies are needed to clarify the pro-tumor and anti-tumor functions of NETs in glioma and BMs.

Macrophages are the major infiltrating immune cells (up to 30% tumor mass) in the brain TME (190). Thus, there was increased focus on macrophages in the brain TME in the past few years. However, one study reported that neutrophils infiltrate the human glioma and the degree of infiltration was markedly correlated with tumor grade (191). However, the mechanism of neutrophil recruitment and the role of neutrophils in tumor growth have not been elucidated. Most studies on neutrophils and brain tumors have focused on the impact of these cells on the response to anti-angiogenic therapy and vascularization (a hallmark of high-grade glioma). Enhanced neutrophil infiltration into tumor tissue is associated with acquired resistance to the vascular endothelial growth factor antibody (bevacizumab) and high glioma grade at advanced stages (191, 192). Preclinical studies have demonstrated that neutrophils contribute to glioblastoma progression by supporting the expansion of the glioma stem cell pool through S100 protein-dependent mechanism (192). S100 proteins are associated with secondary dissemination, especially the dissemination of breast cancer (193). S100A8 and S100A9, which are upregulated in the pre-metastatic brain, promote the recruitment of neutrophils and subsequently promote metastatic seeding (194). The brain may be aberrantly exposed to pathological inflammation associated with the tumor that influences disease progression. Therefore, future studies must focus on the mechanisms of neutrophil infiltration in the brain TME.

## Neutrophil as a Therapeutic Target and Potential Combination of Neutrophil-Targeting With Other Cancer Therapies

**Figure 3:**
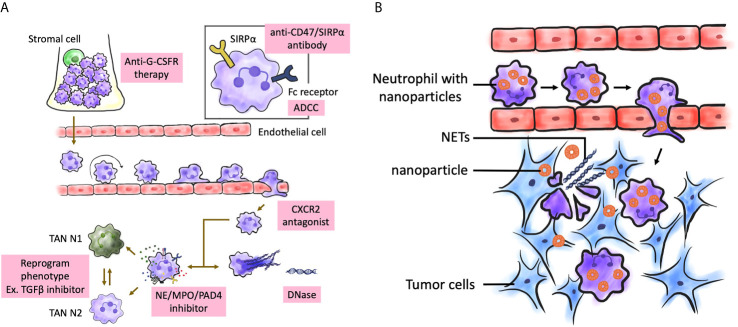
Possible treatment targeting neutrophils in brain tumors. **(A)** Several aspects of neutrophil biological function may be therapeutically targeted from the maturation process to effector functions. The targeting aims are to enhance or inhibit neutrophil function, or to restore normal neutrophil function. Enhancement can be achieved by inhibiting signal-regulatory protein α (SIRPα). Inhibitors of CXCR1, CXCR2 can be used to block neutrophil activation and migration. NE, MPO, and PAD4 inhibitors can be used inhibit NETosis. **(B)** Neutrophils can be nanocarrier to mediate anticancer nanoparticle drug delivery. After surgical resection of brain tumors, inflammatory signals were amplified at post-resection sites, which attracted neutrophils with nanoparticles to migrate into infiltrating brain tumors along the chemotactic gradient, and results in disruption of the neutrophils and release of NETs. That renders a concomitant release drug into tumor microenvironment and produce antitumor effect. NETs were also released on excessive activation by inflammatory cytokines. G-CSFR, G-CSF receptor; MPO, myeloperoxidase; NE, neutrophil elastase; NET, neutrophil extracellular trap; PAD4, peptidylarginine deiminase 4; TAN, tumor-associated neutrophil.

Traditionally, neutrophils were not considered to have a critical role in the TME. However, recent studies have demonstrated that (195–197) neutrophils play a major role in the pathogenesis of cancer, including tumor initiation, development, and progression. Furthermore, several studies have suggested that the presence and phenotype of neutrophils in the TME determine the outcomes of traditional and novel therapies, such as ICIs (198, 199). Most studies indicate that neutrophils promote tumorigenesis (167, 200). Therefore, neutrophils can be directly targeted to inhibit their recruitment, activation, or phenotype reprograming. Recently, the concept of targeting TANs has been proposed by several studies. The polarization of neutrophils (pro-tumor or anti-tumor) in cancers must be characterized to improve the efficacy of therapeutic modalities. Therefore, the development of next-generation immunotherapies is an important topic of research (201). Various approaches have been developed to therapeutically target neutrophils, including strategies to enhance, inhibit, or reprogram the neutrophil phenotype. Clinical trials on TGFβ pathway inhibitors and PDE5 inhibitors for glioma and BMs are ongoing. However, there are several challenges and controversies for using these strategies. For example, Galunisertib, a novel TGFβ receptor 1 kinase inhibitor, is currently undergoing clinical trials as a monotherapy or in combination with lomustine chemotherapy in two clinical trials involving patients with recurrent glioblastoma (NCT01582269 and NCT01682187) and those undergoing temozolomide-based chemoradiotherapy (NCT01220271). Here, we have listed some therapeutic mechanisms that potentially recruit, activate, inhibit, or modulate the phenotypes of neutrophils in the TME, which are currently being investigated in cancers of humans or animals (**Figure 3A**, **Table 1**).

**Table 1:** Agents with putative effects on neutrophils in patients with cancer.

Target	Effects on neutrophils	Agent	Study object
**TGFβ pathway inhibitor**	Promote the development of neutrophils with an antitumor phenotype (165)	Galunisertib (a TGFβR1 kinase inhibitor)	Humans (NCT02734160, NCT01582269, NCT01682187, NCT02452008)
		Fresolimumab (an anti- TGFβ monoclonal antibody)	Humans (NCT02581787)
**CD47-SIRPα inhibitor**	Delay the transmigration of neutrophils to tumor tissues, thus inducing macrophage-mediated phagocytosis of tumor cells (202, 203)	Hu5F9-G4	Humans (NCT02216409)
		IBI188	Humans (NCT03717103)
		CC-90002	Humans (NCT02367196)
**TRAIL-R agonist**	Triggers neutrophil apoptosis and clearance from tissues by targeting TRAIL-Rs expressed on neutrophils (204, 205)	Mapatumumab	Humans (NCT01088347)
		AMG 951	Humans (NCT00508625)
		TRM-1	Humans (NCT00092924)
**ACKR2**	a novel immune checkpoint that regulates neutrophil differentiation, mobilization to tumor tissues and anti- metastatic activity in animal study (206)	–	–
**Chemokine signaling**			
CXCR1/CXCR2	Inhibit neutrophil recruitment to the tumor; attenuate granulocytosis, neutrophil recruitment and vascular permeability by inhibiting the CXCR2 chemotactic axis (207, 208)	SX-682	Humans (NCT03161431)
		Raparixin	Humans (NCT02370238, NCT02001974)
CCR5	Inhibit both the release of immature neutrophils from bone marrow and their recruitment to the tumor (209, 210)	Maraviroc	Humans (NCT03274804, NCT01736813)

### Combining Neutrophil-Targeting Therapy With Other Anticancer Therapies

Several chemotherapies are reported to cause neutropenia and consequently eliminate neutrophils from the TME. However, the effect of these chemotherapies on the neutrophil phenotype modulation and the subsequent implications in treatment efficacy are largely unknown. These patients are at an increased risk of opportunistic infections (211, 212). Furthermore, neutrophils are a key mediator of the efficacy, clinical value, and toxicity of these therapies in patients receiving ICIs (213). However, limited studies have focused on the effects of drugs on cancer-related neutrophil phenotypes. A critical assessment of the most optimal combination therapy strategies is key for the successful clinical implementation of neutrophil-targeting approaches. Various mechanistic studies performed in clinically relevant mouse tumor models have addressed the impact of neutrophils on the efficacy of anticancer therapies.

#### Chemotherapy

Neutropenia is a common “adverse effect” of chemotherapy (214). In the clinics, recombinant G-CSF and GM-CSF are commonly prescribed to increase neutrophil counts and reduce the risk of infection as they promote the release of neutrophils from the bone marrow (22, 215). However, the effects of G-CSF or GM-CSF on human neutrophil phenotypes are unclear. Contradictory findings have been reported for the pro-tumor and anti-tumor activities of the recruited neutrophils.

*In vitro* studies on neutrophils isolated from the peripheral blood of patients who received G-CSF have revealed contradictory findings on neutrophil function, including phagocytosis, oxidative burst, bacterial killing, and chemotaxis (216). Filgrastim (non-glycosylated G-CSF) and lenograstim (glycosylated G-CSF) exhibited differential effects on the chemotaxis and morphology of circulating neutrophils isolated from patients with non-Hodgkin lymphoma (217). Neutrophils from patients who received lenograstim exhibited impaired chemotaxis. In contrast, neutrophils isolated from patients who received filgrastim had a morphology that suggested enhanced activation and upregulated expression of integrin β2. G-CSF and GM-CSF, which are reported to exert pro-tumor and anti-tumor effects, can affect both tumor and immune cells (218–222). Previous studies have reported that G-CSF induces the phagocytic and antibacterial activities of neutrophils (223) and promotes ROS production upon stimulation (224). However, the phenotypic modulation of TANs after treatment with G-CSF and GM-CSF is currently under investigation.

#### Radiotherapy

Radiotherapy is one of the most important treatment modalities for cancer. Several studies on animal models have demonstrated that radiotherapy activates both the adaptive and innate immune responses through the release of antigens, Toll-like receptor ligands, and pro-inflammatory cytokines from tumor cells. This promotes the recruitment of myeloid cells, such as macrophages, dendritic cells, and neutrophils, and induces T cell-mediated immunogenic cell death (225–227). In preclinical models, radiotherapy induces sterile inflammation with rapid and transient infiltration of neutrophils into the tumors (228). These newly recruited neutrophils produce increased amounts of ROS and induce apoptosis in the tumor cells. Recent studies have suggested that the baseline blood neutrophil count is correlated with the survival of patients with different cancers after radiotherapy (229–232). However, limited studies have examined the effects of radiation on neutrophil phenotypes in patients with cancer. Clinical studies have demonstrated that radiotherapy can initiate a response outside the local radiation field (which is known as the “abscopal effect”) and this is correlated with enhanced recruitment of immune cells (233–236). Based on these observations, the combination of radiotherapy and immunotherapy or GM-CSF may improve the clinical outcomes of patients with cancer (237–239). The effects of radiotherapy on human neutrophils are unknown (229).

#### ICIs

ICIs, such as anti-CTLA-4 and anti-PD-1 antibodies, exhibited satisfactory therapeutic efficacy in several patients with advanced-stage cancers, especially in patients with melanoma. Although not all patients benefit from these agents, ICIs are frequently used as first-line therapies in patients with other cancers (240, 241) exhibiting upregulated expression of PD-1, PD-L1, and/or CTLA-4. Several studies have reported the effects of ICIs on the TME in mouse models (242) and patients with cancer (243–245). However, the effects of ICIs on intratumoral neutrophils remain unclear.

In a study published in 2017, changes in intratumoral immune cell subpopulations were investigated in patients with melanoma after treatment with the anti-PD-1 antibody nivolumab (243). The number of intratumoral neutrophils was not markedly different between patients who benefited from nivolumab and those who did not benefit from nivolumab although the intratumoral neutrophil counts varied between the two groups. Several studies have suggested a correlation between PD-L1 expression on neutrophils and an immunosuppressive phenotype. For example, PD-L1+ neutrophils are reported to suppress T cell function and promote disease progression in patients with gastric cancer (199). This suppressive effect may be reversed upon inhibition of PD-L1. The expression levels of PD-L1 in the intratumoral and peritumoral neutrophils were upregulated when compared with those in circulating neutrophils in patients with hepatocellular carcinoma (198). This suggested that TANs exert strong immunosuppressive effects in these patients and that PD-L1+ neutrophils are potential targets of anti-PD-1 and/or anti-PD-L1 antibodies.

### Targeting NETs

NETs, which are involved in antimicrobial immunity, autoimmune conditions, cardiovascular diseases, and tumor progression (66, 198, 246), are a potential therapeutic target for cancer. In several cases, NET formation is dependent on the activity of NADPH oxidase. Thus, the inhibition of NADPH oxidase can alter NETosis (175). Superoxide can be transformed into hydrogen peroxide, which can activate azurophilic (primary) granule proteins, such as neutrophil elastase or MPO (175) and consequently promote the nuclear translocation of some molecules. In the nucleus, neutrophil elastase promotes nuclear decondensation. MPO upregulates the activity of neutrophil elastase. Hence, neutrophil elastase and MPO can serve as therapeutic targets for NET-associated disorders (247, 248). Neutrophil elastase inhibitors are effective in patients with bronchiectasis. Currently, studies on the therapeutic potential of neutrophil elastase inhibitors for bronchiolitis obliterans are ongoing (249). As PAD4 is critical for NET formation, it is a potential therapeutic target for NET-mediated diseases (250–252). The effect of PAD inhibitors on human patients has not been examined. Previous studies have targeted DNase I to alleviate NET-mediated pathology (253–256). Therefore, DNase I-mediated degradation of NETs can be a potential therapeutic strategy for cancers with NET involvement.

## Neutrophil as a Nanocarrier

Surgical resection is the major therapeutic strategy for brain tumors (257). However, the infiltrating tumor cells near the eloquent brain area should not be completely removed to preserve neurological functions (258). Generally, adjuvant chemotherapy is necessary after surgery. However, the efficacy of adjuvant therapy is limited owing to poor drug penetration caused due to various physiological barriers, especially the blood-brain barrier (BBB) and blood-tumor barrier (BTB) (259–261), which contribute to tumor recurrence. Therefore, nanoparticle-based drug delivery systems (NDDSs) are used for enhanced targeting of the tumor (262–265). NDDSs utilize active targeting ligands or passive leakage of tumor vasculature (266–269). However, the efficacy of these NDDSs for postoperative glioma treatment is poor due to a low half-life of the nanoparticles in the circulation, insufficient intratumoral drug accumulation, and severe systemic toxicity.

Recently, cell-based drug delivery systems are considered powerful bioinspired drug delivery platforms for glioma (269–274). Vectorization of therapeutic agents using endogenous cells has been proposed as a potential strategy for targeted drug delivery to the brain (275–277). Neutrophils, which play a critical role in immune responses, can be activated within the vasculature. The activated neutrophils move along chemotactic gradients toward the inflammatory sites and eliminate the pathogens by phagocytosis (278–280). Additionally, neutrophils can cross the BBB/BTB and infiltrate the tumor mass (191, 281–283). TANs, which are distributed in the glioma region (284), promote the continuous recruitment of the circulating neutrophils (201). Surgical tumor excision leads to local brain inflammation with the release of inflammatory factors, such as IL-8 (285, 286) and TNF-α (287, 288) that activate the migration of neutrophils to the inflamed region of the brain (278). The amplification of inflammatory signals supports enhanced targeting of brain tumors.

Therefore, neutrophils could be explored as “Trojan horses” to carry concealed drug cargoes to diseased brain areas (**Figure 3B**). Zhang et al. demonstrated that the physiological activity of neutrophils carrying PTX liposomes was not affected and that these neutrophils migrate to the inflamed brain tumor. These neutrophils improved the survival of postsurgical glioma-bearing mice (289). Traditional nanoparticles passively target the tumor site [which is known as the enhanced permeability and retention effect (290)] or actively target the tumor site through ligand-receptor interactions (291). In contrast, the neutrophil-mediated drug delivery system can recognize postoperative inflammatory signals, such as IL-8 and CXCL1/KC (74, 292) and spontaneously deliver chemotherapeutics to infiltrating glioma cells. The aberrant activation of neutrophils by the upregulated inflammatory cytokines in the inflamed brain results in the disruption of neutrophils and promotes the release of NETs (293) with concomitant release of liposomes to deliver PTX into the remaining infiltrating tumor cells. We believe this strategy will offer new opportunities to explore endogenous immunocytes as drug delivery vehicles. In the future, neutrophils harvested from humans can be used to deliver drugs in clinics.

Several challenges associated with nanoparticle delivery systems must be addressed before translation into a human clinical model. The extraction of neutrophils from patients before intracranial implantation may pose an additional risk and delay surgery. The bioactivity of PTXCL within neutrophils *in vivo* remains unclear. The PTX resistance of glioma cells (as evidenced by the poor efficacy of PTX-CL/NE treatment) suggests that combination treatment must be considered to attain optimal therapeutic efficacy. However, neutrophil-mediated DDSs targeting the glioma-initiating stem cells in the perivascular niche can have potent therapeutic benefits (294, 295).

## Conclusion and Perspective

The role of neutrophils in cancer biology and their potential as therapeutic targets have been widely recognized. Recent studies have demonstrated the important biological roles of neutrophils. The complex roles of neutrophils in cancer include their ability to exert pro-tumor or anti-tumor activities and to exhibit various polarization phenotypes. The elucidation of the interaction of neutrophils in cancer can aid in the development of novel therapeutic interventions. For example, targeting TANs and/or circulating neutrophils can be potential next-generation immunotherapies. However, further studies are needed to examine the exact roles, recruitment pathways, subpopulations, and mechanisms of action of TANs to develop targeted therapeutic approaches. Additionally, the role of TANs in the brain TME is not clear although the extent of neutrophil infiltration is correlated with glioma grades. Thus, TANs in the brain TME are a potential therapeutic target for brain cancer. Additionally, TANs can improve the efficacy of chemotherapy or immunotherapy.

There are contradictory reports on several characteristics of neutrophils, including lifespan, transcriptional activity, roles in cancer, and subpopulation types. Additionally, neutrophils may escape therapeutic interventions because of their exceptional turnover and unexpected plasticity, especially in cancer. However, recent understanding of neutrophil biology has revealed that precise therapeutic interventions may provide therapeutic benefits without detrimental side effects. In particular, NETs are a key therapeutic target (296). Future studies must focus on small-molecule and biological therapeutics that can regulate the neutrophil compartment to promote the activation, inhibition, or depletion of neutrophils. Additionally, antibody-mediated delivery of small molecules can be a potential therapeutic strategy. Recent studies have suggested the potential applications of neutrophils as drug-trafficking cells (289, 297). Neutrophil-derived molecules, such as granule proteins and peptides may also be used as therapeutic agents under certain conditions. In summary, these findings indicate the potential of targeting neutrophils in human diseases.

## Author Contributions

Y-JL, ML, and T-LH wrote and revised the manuscript. K-CW and P-YC consulted and revised the manuscript. Y-JL drew the figures. ML and T-LH initiated the concept and supervised the writing. All authors contributed to the article and approved the submitted version.

## Funding

This study is sponsored by Chang Gung Memorial Hospital (grants CMRPG3H1141). The sponsor had no role in the design or conduct of this research.

## Conflict of Interest

The authors declare that the research was conducted in the absence of any commercial or financial relationships that could be construed as a potential conflict of interest.

## Publisher’s Note

All claims expressed in this article are solely those of the authors and do not necessarily represent those of their affiliated organizations, or those of the publisher, the editors and the reviewers. Any product that may be evaluated in this article, or claim that may be made by its manufacturer, is not guaranteed or endorsed by the publisher.
